# A Measurement-Based Frame-Level Error Model for Evaluation of Industrial Wireless Sensor Networks

**DOI:** 10.3390/s20143978

**Published:** 2020-07-17

**Authors:** Yun-Shuai Yu, Yeong-Sheng Chen

**Affiliations:** 1 Department of Computer Science and Information Engineering, National Formosa University, Yunlin 632301, Taiwan; yys@nfu.edu.tw; 2 Department of Computer Science, National Taipei University of Education, Taipei 106320, Taiwan

**Keywords:** IWSNs, error models, IEEE 802.15.4, second-order Markov chain, OpenWSN, transmission reliability

## Abstract

Industrial wireless sensor networks (IWSNs) are a key technology for smart manufacturing. To identify the performance bottlenecks in an IWSN before its real-world deployment, the IWSN must first be evaluated through simulations using an error model which accurately characterizes the wireless links in the industrial scenario within which it will be deployed. However, the traditional error models used in most IWSN simulators are not derived from the real traces observed in industrial environments. Accordingly, this study first measured the transmission quality of IEEE 802.15.4 in a one-day experiment in a manufacturing factory and then used the measurement records to construct a second-order Markov frame-level error model for simulating the performance of an IWSN. The proposed model was incorporated into the simulator of OpenWSN, which is an industrial WSN implementing the related IEEE and IETF standards. The simulation results showed that the proposed error model improved the accuracy of the estimated transmission reliability by up to 12% compared to the original error model. Moreover, the estimation accuracy improved with increasing burst losses.

## 1. Introduction

Industrial wireless sensor networks (IWSNs) play a key role in smart manufacturing by enabling a wide range of monitoring, control and optimization processes. In a typical IWSN, the operating parameters of a machine in the factory, e.g., the vibration frequency, pressure, flow rate, coolant level, and so on, are measured by one or more sensors and the sensor signals are then transmitted wirelessly to a gateway, from which they are passed to a remote server via a wired or wireless network for further processing and/or storage. Typically, the server performs online calculations on the sensed data using domain knowledge and transmits appropriate control commands back to the factory to control the corresponding actuators, provided that the calculation results satisfy certain predefined conditions. For example, the server may instruct the machine to close a valve or reduce the conveyor speed. The remote server may also analyze the sensed data offline, e.g., by inputting the manufacturing parameters and product yield data into a Deep Neural Network (DNN) in order to derive the optimal manufacturing parameters, for example.

There are two international standards for the IWSN protocol stack in industry at present, namely WirelessHART [1], established by the HART Communication Foundation (HCF), and ISA 100.11a [2], developed by the International Society of Automation (ISA). In both standards, the data transmissions in the physical layer are performed using IEEE 802.15.4-2006 [3], while media access is achieved using Time Division Multiple Access (TDMA), and routing at the network layer is conducted using Graph Routing. Academic researchers and industrial organizations, such as IEEE and IETF, have proposed many communication protocols and standards for IWSNs. Many of these protocols are implemented in OpenWSN [4], an open-source project using IEEE 802.15.4-2006 in the hardware layer (like WirelessHART and ISA 100.11a) and Time-Slotted Channel Hopping (TSCH) technology in IEEE 802.15.4e [5] in the lower part of the Media Access Control (MAC) layer. In addition, the higher part of the MAC layer adopts the Internet Protocol version 6 (IPv6) over the Time Slotted Channel Hopping mode of IEEE 802.15.4e (6TiSCH) Minimal Scheduling Function (MSF) and the 6TiSCH Operation Sublayer (6top) Protocol (6P) formulated by the 6TiSCH working group of the IETF. The network layer adopts protocols such as IPv6, Internet Control Message Protocol version 6 (ICMPv6), IPv6 over Low-Power Wireless Personal Area Networks (6LowPAN) and Routing Protocol for Low-Power and Lossy Networks (RPL) formulated by the IETF, while the transport layer uses User Datagram Protocol (UDP) and Constrained Application Protocol (CoAP), also formulated by the IETF. The application layer is left to the user for customization.

The transmission reliability, delay and lifetime of an IWSN are all critically dependent on its design. However, an IWSN is often composed of hundreds or even thousands of sensing nodes. Thus, optimizing the IWSN design in-situ using experimental methods is extremely challenging, if not impossible. Accordingly, before the actual deployment of the IWSN, it is desirable to evaluate the function and effectiveness of the network through systematic simulations using a simulator model as close as possible to that of the environment in which the network will be deployed. Among all the measures of the IWSN performance, the transmission reliability is one of the most important, and is defined as the probability of successful delivery of the sensed data from the sensing nodes to the gateway used to route the data to the remote server. In real-world environments, the transmission of frames via the wireless medium may fail for various reasons, including noise interference, low signal energy due to multipath attenuation, and so on. To simulate such transmission failures, it is necessary to employ an error model which accurately characterizes the errors associated with the real wireless environments. In particular, simulations on the cases with lots of transmission failures are important for applications requiring dependable operations, e.g., gas metering applications. The OpenSim simulator [6] in OpenWSN uses an independent error model (also known as the Bernoulli error model), which assumes that the frame transmission success has a fixed probability (generally referred to as the Frame Delivery Ratio (FDR)). However, the transmission quality of wireless links is affected by many factors in the field environment and frequently changes over time. Consequently, the simple Bernoulli error model limits the ability of the OpenWSN simulator to accurately evaluate the transmission performance of an IWSN.

To address this problem, the present study derives a frame error model which more accurately represents the wireless conditions in a real-world factory environment by measuring the transmissions in a machine-intensive factory over a period of almost 24 h and then using the measurement records to construct a frame-level second-order Markov model as an error model. Note that the proposed error model is mainly suitable for the industrial environments where there are only IEEE 802.15.4 devices operating in 2.4 GHz Industrial, Scientific and Medical (ISM) band (that is, it will be inappropriate to apply our model in the factory environment with devices supporting IEEE 802.11.). It is shown that the Cumulative Distribution Functions (CDFs) of the number of consecutively received correct frames (i.e., the correct-frame burst length) and the number of consecutively received error frames (i.e., the error-frame burst length) synthesized by the proposed model are very close to those of the original records. Moreover, the simulation results show that the proposed model improves the estimation accuracy of the transmission reliability by up to 12% compared to that achieved using the original independent error model in OpenWSN.

The remainder of this paper is organized as follows. Section 2 briefly reviews the related work in the literature. Section 3 describes the data collection methods employed in the present study and gives the preliminary analysis results. Section 4 describes the proposed error model and verifies its correctness. Section 5 analyzes and explains the overestimation errors of the independent error model in OpenWSN for the transmission reliability. Section 6 presents and discusses the simulation results obtained using the proposed error model. Finally, Section 7 provides some brief concluding remarks.

## 2. Related Work

In general, error models for IWSNs can be categorized into two main types, namely bit-level error models and frame-level error models. Models of the former type consider each bit of a frame to be either erroneous or correct, and assume that a frame is transmitted successfully if no bit is wrong. By contrast, models of the latter type judge the entire frame directly as either erroneous or correct. Generally speaking, bit-level error models provide subtler channel-state information than frame-level models, whereas the frame-level models offer better scalability due to their greater simplicity.

Nobre et al. [7,8,9] developed a WirelessHART communication module for Network Simulator 3 (NS-3) [10] based on a bit-level two-state Markov chain error model designated as the Gilbert/Elliot model. Remke et al. [11] modeled WirelessHART networks with bit failures using a Binary Symmetric Channel (BSC) model [12] and link failures using a two-state Markov chain. Barac et al. [13] analyzed the bit- and symbol-error nature of IEEE 802.15.4 transmissions in actual industrial sites and then employed the collected error traces to evaluate the performance of a lightweight Reed-Solomon (15, *k*) block code.

Gao et al. [14] adopted a frame-level two-state Markov error model to evaluate the Quality-of-Service (QoS) performance of IEEE 802.15.4 MAC under bursty channel errors. Petrova et al. [15] analyzed the performance of IEEE 802.15.4 based on Received Signal Strength Indicator (RSSI), Frame Error Rate (FER) and run length distribution measurements obtained in indoor and outdoor environments. It was shown that the independent and two-state Markov error models both provided an adequate modeling performance for short transmission distances, but the two-state Markov model slightly outperformed the independent model over longer distances. Wijetunge et al. [16] used a three-dimensional discrete-time Markov chain model to analyze the IEEE 802.15.4 MAC protocol with Acknowledgement (ACK) frame transmission.

Iqbal and Khayam [17] conducted transmission experiments in a two-story building for transmission distances of 5 to 12 m, a frame size of 20 bytes and a transmission rate of 10 frames per second. The transmission records were then used to construct a two-level error model consisting of a two-state Markov model in the first level for predicting the error probability of the frames, and a third-order Markov model in the second level for-predicting the error probability of each bit in any frames flagged in the first level. However, since the transmission records were not collected in factories, there is no guarantee that the two-level error model can be applied in IWSN environments. Ilyas and Radha [18] measured the transmission quality in office, home and outdoor environments over channel 26 (2479–2481 MHz) for a transmission power of 0 dBm and a transmitted frame size of 41 bytes. The measurement results obtained for partially lost frames and frames that failed the Cyclic Redundancy Check (CRC) test were then used to construct a a discretized exponential Probability Density Function (PDF) error model. However, as for the study of Iqbal and Khayam [17], the model is not directly applicable to factory environments. Moreover, the experimental measurements did not consider the case where the frames were not received at all.

Striccoli et al. [19] proposed a Markov error model with *K* states to account for the various conditions a lossy wireless channel may undergo. Each state contained two substates, ON and OFF, modeled with probability distributions of Error Free Bursts (EFBs) and Error Bursts (EBs) respectively, where an EFB represents a sequence of consecutive correct frames, while an EB represents a sequence of consecutive erroneous frames. The state of the error model was switched among the substates to generate EFB/EB from the probability distributions of the selected substate. However, the model was trained using data traces collected in laboratories rather than industrial environments. Furthermore, a frame was marked as correct only when the transmitter received acknowledgment of the frame’s correct receipt. Hence, although the traces reflect the characteristics of bi-directional transmissions, they may not be suitable for unidirectional transmissions, such as beacon frame broadcasting.

Guntupalli et al. [20,21] proposed a frame-level error model for error-prone channels with one state in the loss macro-state and three states in the non-loss macro-state. However, the error model was configured according to field measurements [22] collected in an 802.11 Wireless Local Area Network (WLAN) rather than IEEE 802.15.4 WLAN. Valle et al. [23,24] proposed a retransmission scheme for WSNs based on cooperative relays and network coding and evaluated its effectiveness using Objective Modular Network Testbed in C++ (OMNeT++) [25] with a semi-Markov error model [26].

## 3. Transmission Quality Measurement and Analysis

**Figure 1:**
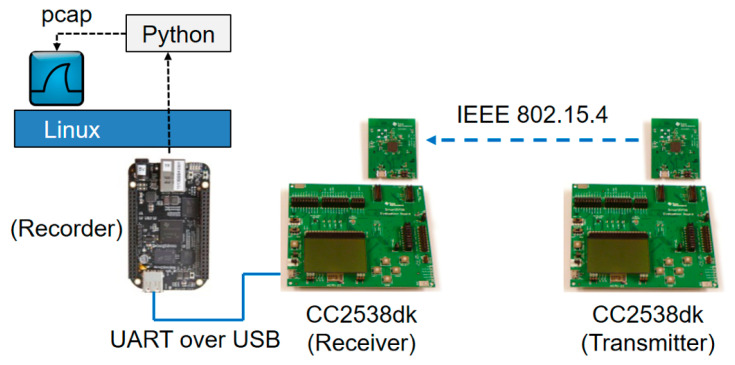
Devices for recording transmission quality.

In this study, the wireless transmission quality in an industrial environment was recorded using the measurement system shown in Figure 1, consisting of a transmitter, a receiver and a recorder. The transmitter and receiver were constructed using Texas Instruments’ CC2538dk development kit [27], incorporating a CC2538 chip with a built-in 2.4 GHz radio frequency (RF) transceiver compliant with IEEE 802.15.4 specifications. The transmitter was installed with Contiki OS 2.7 [28], together with a self-written program for continuously broadcasting signal frames with a frequency of 512 Hz and a length of 23 bytes. Similar to the previous studies in References [17,18], small frame size was adopted in our measurement experiment. However, the present study deliberately adopts a high traffic generation frequency from 10 frames per second to 512 frames per second for extracting the subtler features of the wireless link. In addition, the reasons for transmission using broadcasting technology are three-fold. First, it avoids retransmitting the same frame so that the receiver could better identify the frame loss. Assume that a unicast frame fails and one of its following retransmitted frames was successfully received. In such a case, the receiver cannot determine the number of transmission failures that actually happened. Second, the lack of retransmission mechanism guarantees that the high traffic generation frequency will not be affected. Third, the experimental architecture can be easily extended in the future to include more receivers and recorders without increasing the number of transmitters.

**Figure 2:**
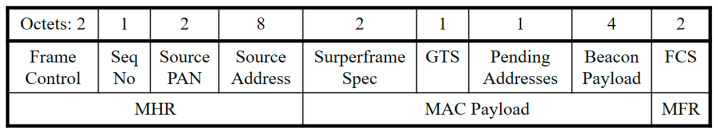
Format of signal frame in trace experiments.

The beacon frame format is shown in Figure 2, in which the beacon payload has the form of a 4-byte positive integer with an initial offset of 0 and an increment of one each time a frame is sent. The abbreviations MHR, MFR, GTS and FCS represent MAC header, MAC footer, guaranteed time slots and frame check sequence, respectively. The receiver was also installed with Contiki OS 2.7 and a self-written receiver program designed to send the received information (e.g., the frame contents at the MAC layer, the RSSI value and the CRC outcome, to the recorder with Universal Asynchronous Receiver-Transmitter (UART) signals over a Universal Serial Bus (USB)).

The recorder was built around a BeagleBone Black [29] single-board microcomputer. The USB signals received by the recorder were restored to UART signals by the Linux kernel embedded in the recorder and input to a Python program via a serial port. The experiment may be interrupted by some unpredictable events, such as power failure or collision caused by the workers. To reduce the impact of such interruptions, the Python program saved the information received each hour in the experimental measurement period to a pcap file (note that the pcap format is supported by many network packet monitoring systems, including Wireshark [30]).

To support the IWSN context considered in the present study, and in particular, to understand the effects of electromagnetic noise generated by the working machines on the transmission quality of IEEE 802.15.4 wireless communications, the measurement process was conducted in a machine-intensive factory in southern Taiwan. To protect trade secrets, the factory prohibits the use of any devices supporting IEEE 802.11 (e.g., access points and smartphones) in the factory. Consequently, the measured traces were free of interference from IEEE 802.11 or Bluetooth networks. Moreover, to avoid disruption of the factory operations, the trace experiment was performed only once, as described below.

**Figure 3:**
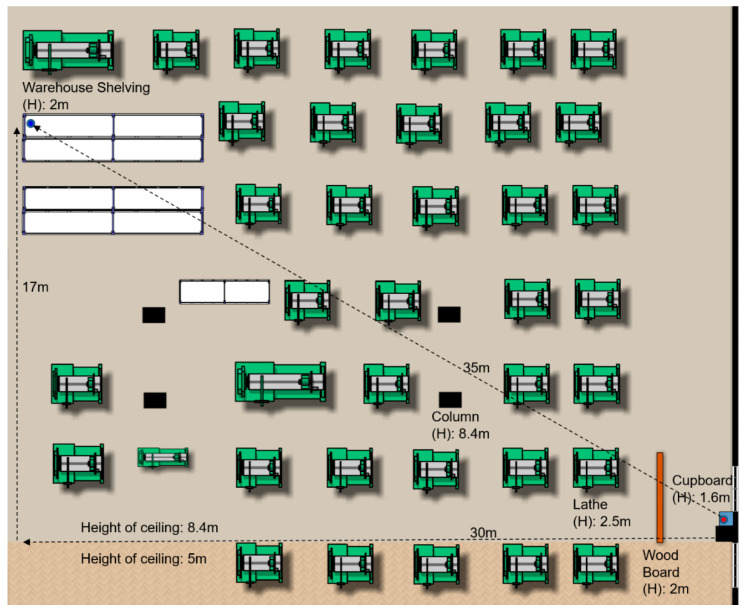
Layout of experimental environment.

Figure 3 illustrates the basic layout of the experimental sensing field with dimensions of approximately 17 × 30 m (length × width). Although the actual dimensions of the field are approximately 100 m in length and width, only the areas related to the experiment are drawn for ease of illustration. The factory had two ceiling heights, namely 8.4 m in the majority of the building (upper part of Figure 3) and 5 m in the remainder (bottom part of Figure 3). The transmitter was located in one corner of the building (see the red dot in Figure 3) and was placed on top of a cabinet with a height of 1.6 m. There were windows behind the cabinet, and hence the transmitter was exposed to sunlight during the afternoon period of the experimental process. The transmission power was set as –1 dBm throughout the entire measurement process and the transmissions were performed using channel 25 (2474–2476 MHz). The receiver was positioned together with the recorder on a shelf with a height of approximately 2 m in the diagonally opposite corner of the building (see the blue dot in the upper-left-hand corner of Figure 3). The receiver and transmitter were separated by a distance of approximately 35 m, but were not in direct line of sight of one another due to the presence of lathe machines on the factory floor with a height of around 2.5 m. In the path between the transmitter and the receiver, except for the static obstacles, there existed lots of dynamic obstacles. The raw and semi-finished materials on the machines in the path were often moved from one machine to another by the workers. Also, some workers might often walk across the path. Thus, the number of obstacles in the transmission path was dynamic. The measurement period commenced at 15:45 on one day and ended at 15:30 the following day. During the experimental period, the lathes operated independently in accordance with their particular work orders. That is, not all of the machines worked together, and none of the machines operated over the entire experimental period. Furthermore, in the factory, the daily work content is roughly the same, although there might be slight differences. That is, a day is just a cycle. Therefore, the data measured in one day could be used to characterize the factory environment. In Reference [18], the authors stated that “We collected error traces of approximately 10 million packets in a way that provides, to the authors’ best knowledge, an unprecedented level of insight into the effects of the wireless channel state on the level of corruption of packets.” By contrast, there were up to 43,728,940 transmission records collected in our experiment. According to our studies and the results presented in Reference [18], this amount of data could be able to provide valuable insights into the quality of the wireless link.

**Figure 4:**
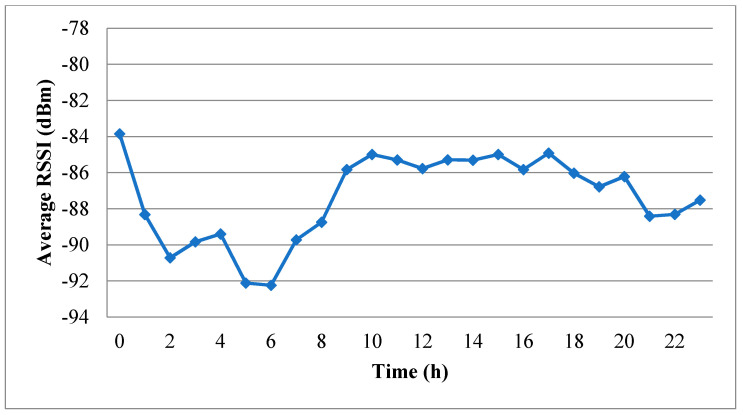
Average Received Signal Strength Indicator (RSSI) value in each hour of the experimental measurement period.

Figure 4 shows the average RSSI values recorded in every hour of the experimental period, where the 0th hour runs from 15:45 to 16:44. It is seen that even though the distance between the transmitter and the receiver remained unchanged, the average RSSI varies significantly over time. In fact, a detailed inspection of Figure 4 shows that the difference between the largest and smallest average RSSI values is more than 8 dBm. The variation in the average RSSI is most likely the result of changes in the indoor temperature, noise interference produced by machines, the blocking or reflection effect of obstacles between the transmitter and the receiver, and so on. The measurement results in Reference [31] show that the RSSI decreases when the temperature increases. Notably, the factory faces the sun in the afternoon and the heat gets trapped inside until night. It might take hours to dissipate the heat after the sunset. Hence, the room temperature went down after 22:00. As a result, the average RSSI from afternoon to midnight is generally low, most probably because of the high room temperature caused by sunlight exposure.

**Figure 5:**
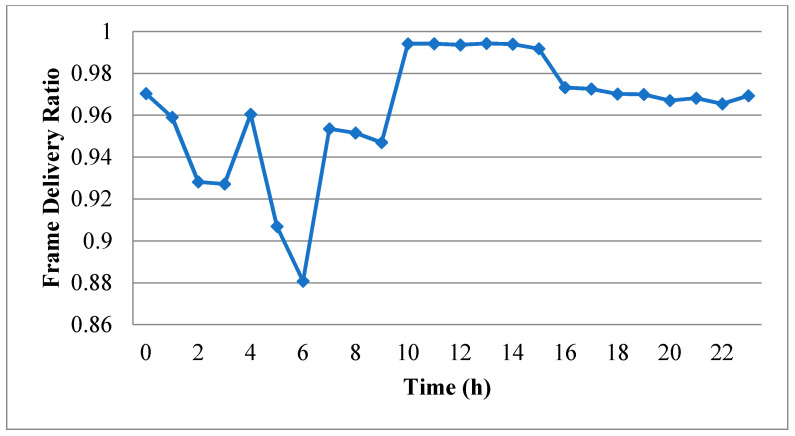
Frame Delivery Ratio (FDR) in every hour of the experimental measurement period.

Figure 5 shows the variation in the FDR over the 24 h experimental period. As expected, the tendency of the FDR is very similar to that of the RSSI in Figure 4. The correlation coefficient between the RSSI and the FDR can be calculated as follows:

(1) $$Correl(x,y)=\frac{\sum_{i=0}^{23}\left(x_{i}-\bar{x}\right)\left(y_{i}-\bar{y}\right)}{\sqrt{\sum_{i=0}^{23}{\left(x_{i}-\bar{x}\right)}^{2}}\sqrt{\sum_{i=0}^{23}{\left(y_{i}-\bar{y}\right)}^{2}}},$$

where *x_i_* is the average RSSI value in a certain hour *i*, $\bar{x}$ is the average of *x_i_* (*i* = 0, 1, …, 23), *y_i_* is the FDR in a certain hour *i*, and $\bar{y}$ is the average of *y_i_* (*i* = 0, 1, …, 23).

The correlation coefficient was found from Equation (1) to have a value of 0.857. In other words, the FDR is extremely sensitive to the RSSI. Thus, when constructing the error model using the measured traces, the parameters in the model should be trained using the transmission records acquired during time intervals with a similar RSSI value. For example, the measurement data obtained in the fifth and sixth hours are suitable for training the model together since the average RSSI values are around −92 dBm in both hours. Similarly, the data from the fourth, seventh and eighth hours are also suitable for training the model together since they all have average RSSI values of around −89 dBm. Finally, the data from the ninth to eighteenth hours can also be used to train the model together since their average RSSI values are all around −85 to −86 dBm. In the subsequent simulations, the error model can then switch among the parameters trained using different sets of trace data in accordance with a switching policy formulated in advance by the IWSN investigator.

**Figure 6:**
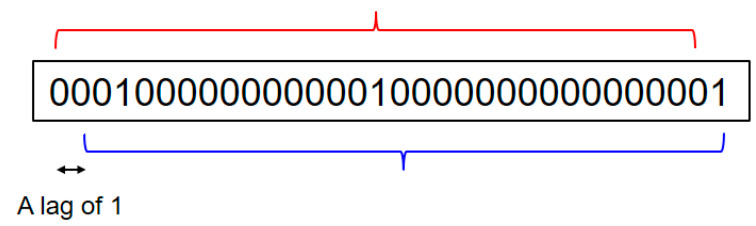
Illustrative example of binary record.

Referring to Figure 5, the FDR has its lowest value in the sixth hour of the experimental period (i.e., 21:45–22:44). In other words, the transmission quality within the factory is particularly poor during this period. As a result, the transmission records acquired in the sixth hour were scrutinized particularly carefully. In particular, the records in the corresponding pcap file were converted into binary records arranged in chronological order from left to right, as shown in Figure 6, in which values of 0 and 1 indicate that the frame transmitted at the particular time was successfully and unsuccessfully received, respectively. Note that failure cases were recorded if the corresponding frame was partially lost, or the length of the received frame was the same as that of the transmitted frame, but the received frame failed to pass the cyclic redundancy check.

**Figure 7:**
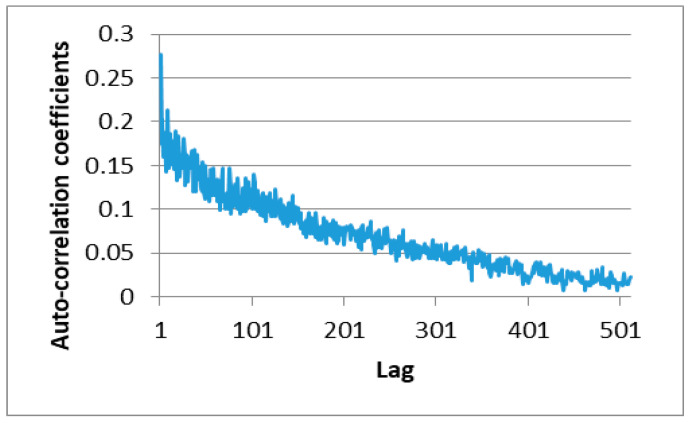
Autocorrelation coefficients of received data in first minute (21:45) of trace obtained in the sixth hour.

**Figure 8:**
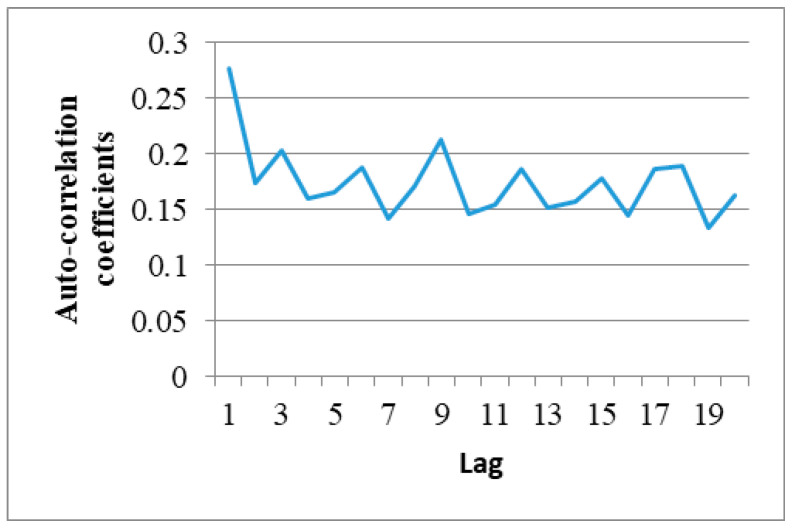
Autocorrelation coefficients of received data in time interval of 20/512 s within first minutes (21:45) of trace obtained in the sixth hour.

Equation (1) was then applied to determine the autocorrelation between the data corresponding to the binary record of the first minute of the sixth hour (21:45). Note that in implementing Equation (1), *x* and *y* were taken as two values in the selected trace with a certain time lag between them. For example, referring to Figure 6, the data enclosed within the lower curly bracket lag those within the upper curly bracket by a notional time value of 1. Figure 7 shows the autocorrelation coefficients computed for the data in the first minute of the selected trace. As described in Section 3, the transmitter used in the present experiments had a broadcast frequency of 512 Hz. In other words, the transmitter broadcasts 512 transmissions each second. As a result, the time lag in Figure 7 has a value of 1/512 s. In general, the results show that the data received at the receiver with a gap of 1 s have almost no correlation with each other. The autocorrelation of the data within the first 0.3 s of the time interval is around 0.1, or more. Figure 8 shows the autocorrelation of the data received over a period of 20/512 s within the first minute of the recorded trace. The above results show that the success or failure of the current transmission does depend on the previous transmission results and mainly depends on the last transmission results. Furthermore, this implies that the collected trace data could be used to train the proposed second-order Markov model, which will be elaborated in the following section.

## 4. Proposed Error Model

The conventional two-state Markov model assumes that the current state is correlated only with the previous state. That is, it is independent of the states before the previous state. However, the autocorrelation coefficients presented above gradually decrease as the time lag increases. Thus, in general, a Markov model with higher orders provides a better representation of the true error situation within the factory environment than one with lower orders. It is noted, however, that the improvement offered by this higher-order model reduces as the order increases. In practice, increasing the order of the Markov model incurs a greater computation cost and larger memory requirement. Thus, determining the optimal order of the Markov model which provides the best tradeoff between the improved realism and the cost is essential. Consequently, the present study deliberately adopts a second-order Markov frame-level error model for evaluating the accuracy of IWSN simulators.

**Figure 9:**
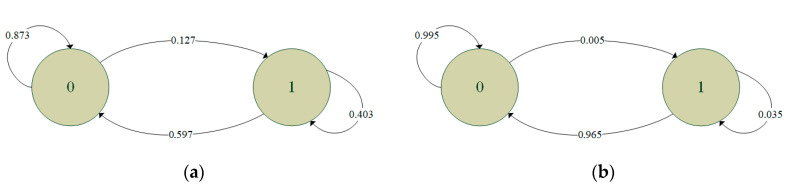
Training results for the two-state Markov model using: (**a**) records in the first minute of the sixth hour, and (**b**) records in the first minute of the tenth hour.

**Figure 10:**
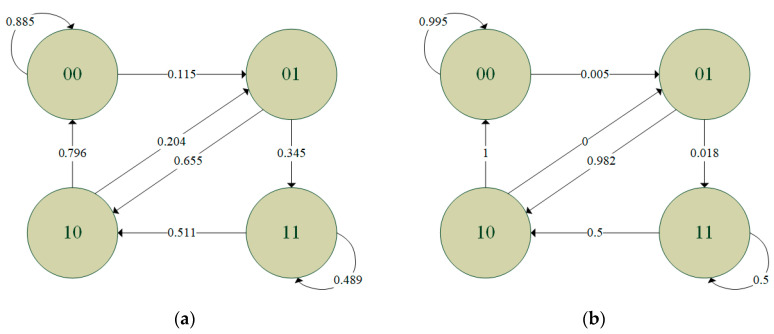
Training results for the second-order Markov model using: (**a**) records in the first minute of the sixth hour, and (**b**) records in the first minute of the tenth hour.

Since the transmission quality in the sixth hour is the worst among all the hours in the experimental period, while that in the tenth hour is the best (see Figure 5), the binary records of the first minutes in these two hours were used to train the independent model, the two-state Markov model and the second-order Markov model. The FDRs of the independent model derived from the two binary records were found to be 0.825 and 0.994, respectively. The training results for the two-state Markov model are shown in Figure 9, where the circles indicate the states and the digits in the circles represent the results of the previous transmission (i.e., 0: successful transmission, 1: failed transmission). In addition, the links between the circles indicate the change from the old state to the new state, where the end without an arrow is the old state and the end with an arrow is the new state. Finally, the numbers attached to the links denote the corresponding transition probabilities. Figure 10 shows the training results obtained for the second-order Markov model (note that the meanings of the symbols in Figure 10 are the same as those in Figure 9 except for the two digits in each circle, where the digit on the right indicates the result of the previous transmission, while that on the left indicates the result of the transmission before the previous one). For ease of discussion, the transition probability in the second-order Markov model is denoted as *pABC*, where *p* is the probability, *AB* is the old state and *BC* is the new state. For instance, the probability of transitioning from state 11 to state 10 is denoted as *p*110. From the results presented in Figure 9 and Figure 10, it is clear that the transition probabilities trained by the two datasets are very different. In other words, the results support the inference above that the parameters in the error model should be trained based on the transmission records obtained during the time intervals with similar average RSSI values.

**Figure 11:**
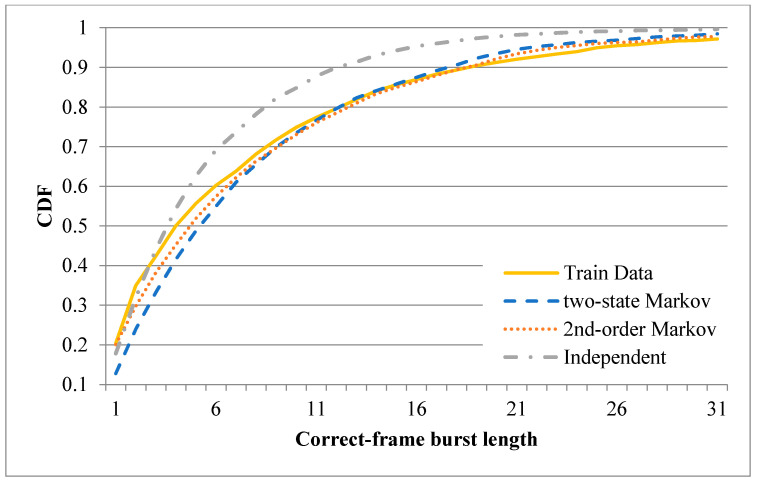
Cumulative Distribution Functions (CDFs) of correct-frame burst length derived using different error models based on recorded trace in the first minute of the sixth hour.

**Figure 12:**
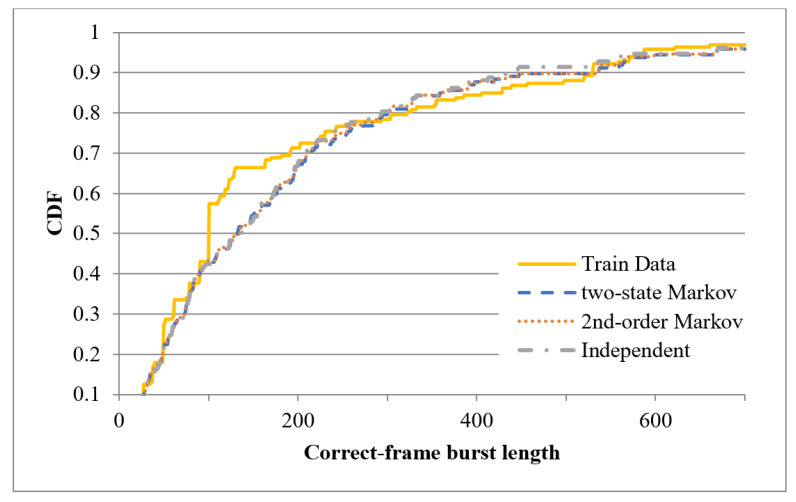
CDFs of correct-frame burst length derived using different error models based on recorded trace in the first minute of the tenth hour.

**Figure 13:**
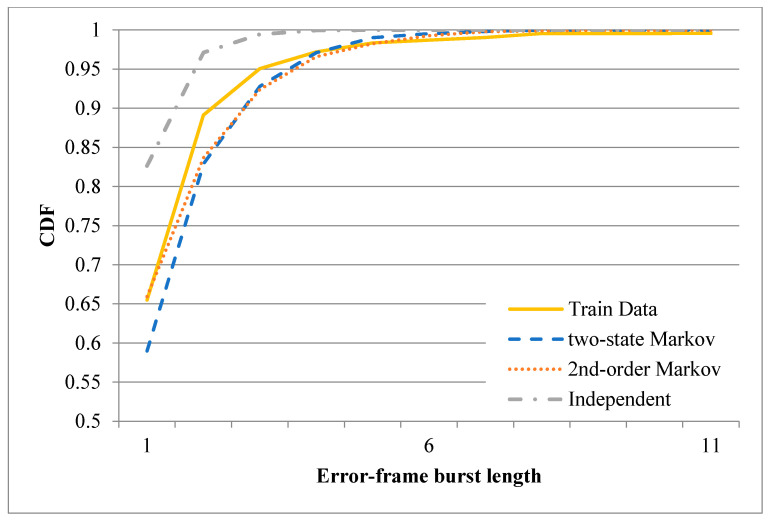
CDFs of error-frame burst length derived using different error models based on recorded trace in the first minute of the sixth hour.

**Figure 14:**
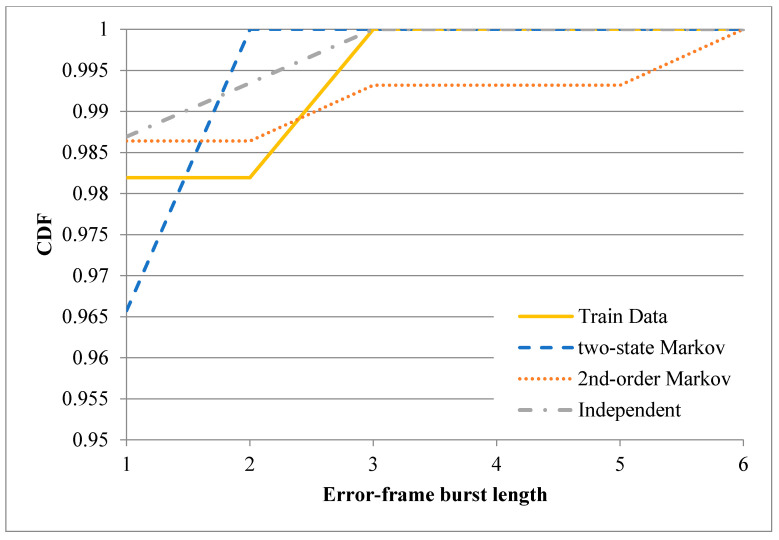
CDFs of error-frame burst length derived using different error models based on recorded trace in the first minute of the tenth hour.

After training the three error models described above, one-minute binary records were synthesized using each of the three models (note that the starting state for the two-state Markov model was set as 0, while that for the second-order Markov model was set as 00). The CDFs of the correct-frame burst length derived from the original records and the three synthesized records are plotted in Figure 11 and Figure 12 for the first minute of the sixth hour and the first minute of the tenth hour, respectively. Figure 11 shows that the CDF of the correct-frame burst length derived using the second-order Markov model is closer to that of the correct-frame burst length derived using the original records than those derived using the independent model or two-state Markov model. However, referring to Figure 12, the three error models have a similar performance. Figure 13 and Figure 14 show the CDFs of the error-frame burst length derived from the original records and the synthesized records for the first minute of the sixth and tenth hour, respectively. It is seen in Figure 13 that the performance of the second-order Markov model is comparable to that of the two-state Markov model and is better than that of the independent model. However, for the records collected in the first minute of the tenth hour, all three models have a similar performance (see Figure 14). This finding is reasonable since the transmissions in the tenth hour have the best quality among all the traces collected in the experimental period. In other words, the trace data in the tenth hour contain only a small number of error-frame bursts for training purposes. Consequently, for all three models, the length of most error-frame bursts is equal to 1. Overall, however, the results presented in Figure 11, Figure 12, Figure 13 and Figure 14 show that the proposed second-order Markov error model outperforms both the independent model and the two-state Markov model when the transmission quality is poor.

As described above, the lack of error-frame bursts in the trace limits the ability of the trained error models to describe the error-frame bursts in the real environment. To address this problem, a further training process was performed using additional experimental traces. In particular, the trace data of the first 2*^n^* minutes of the sixth hour were used to retrain the three models, where *n* is an integer ranging from 0 to 5. The authenticity of the resulting error models was quantified by computing the Kullback-Leibler (K-L) divergence [17] of the synthesized data relative to the original records, i.e.,

(2) $$D\left(p∥q\right)=\sum_{x}p\left(x\right)\log\frac{p\left(x\right)}{q\left(x\right)},$$

where *x* is the correct-frame (or error-frame) burst length, *p* is the PDF of the original records and *q* is the PDF of the synthetic data. The closer to zero the K-L divergence is, the more accurately the error model captures the burstiness of the original records.

**Figure 15:**
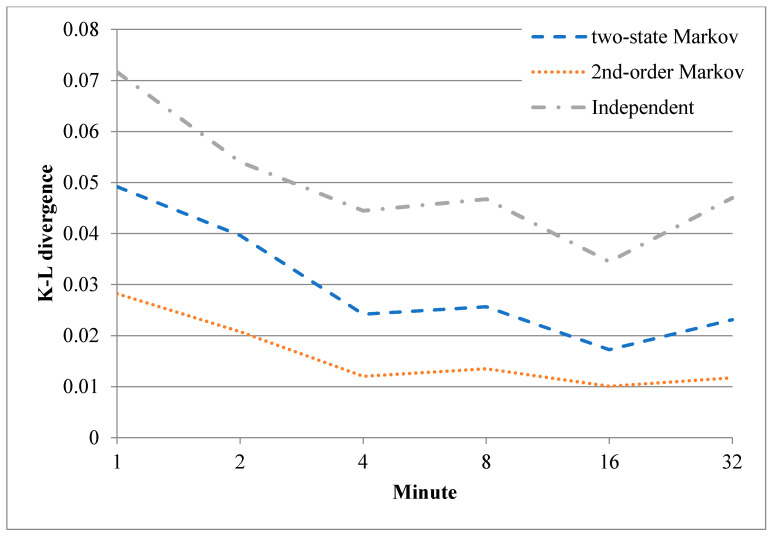
Kullback-Leibler (K-L) divergence between correct-frame bursts of original records and those of synthetic data.

**Figure 16:**
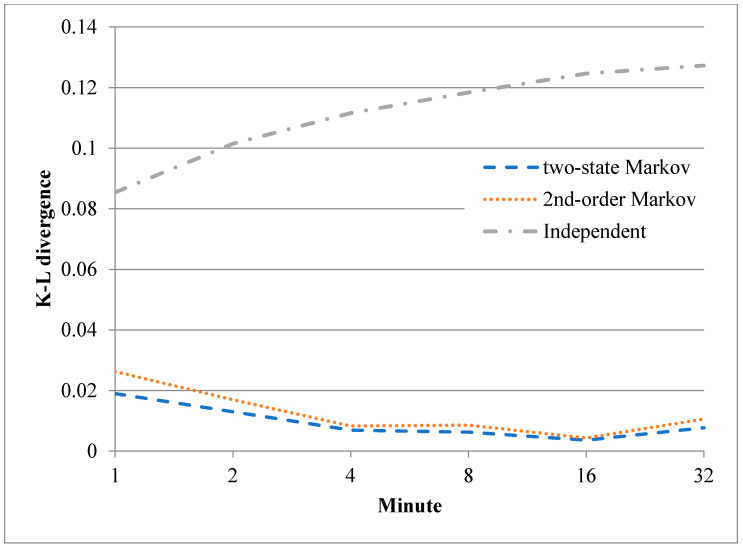
K-L divergence between error-frame bursts of original records and those of synthetic data.

Figure 15 and Figure 16 show the results obtained for the K-L divergence between the correct-frame bursts and error-frame bursts of the original records and those of the synthetic data produced by the three error models, respectively (note that for both figures, the plotted data show the average values obtained over 100 computation processes performed with different synthetic data). As shown in Figure 15, the divergence of all three models reduces (i.e., the authenticity of the models increases) as a greater number of original records are employed in the training process for the correct-frame bursts. A similar tendency is noted for the two Markov models for the error-frame bursts, as shown in Figure 16. However, for both training processes, the divergence improves only very slightly as the length of the training data is increased beyond 4 min. It is additionally noted in Figure 16 that the independent model performs poorly in capturing error-frame bursts in lossy environments.

**Figure 17:**
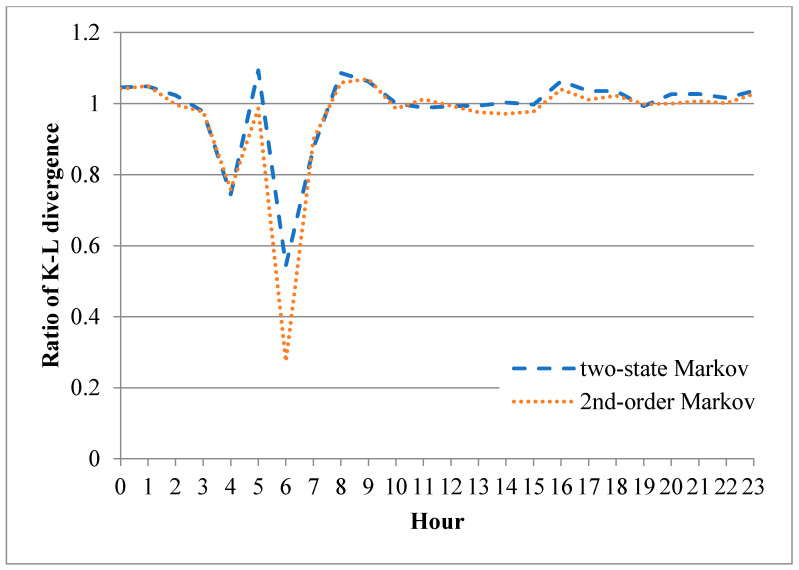
K-L divergence ratios of Markov error models for correct-frame bursts in each hour of the experimental period.

**Figure 18:**
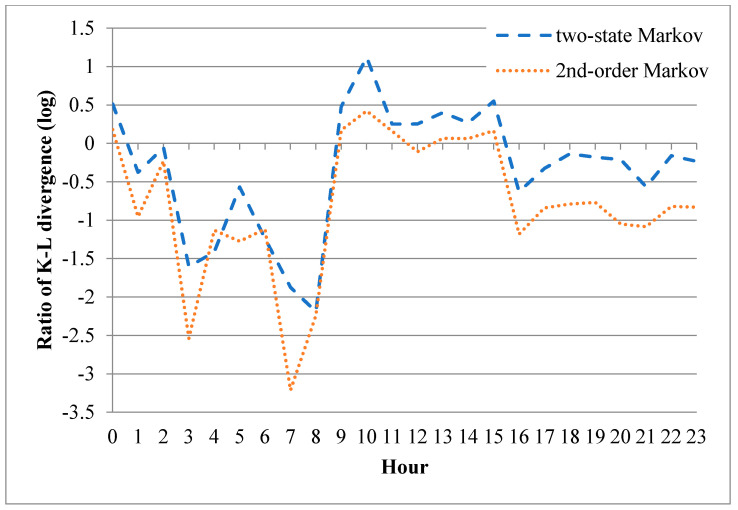
K-L divergence ratios of Markov error models for error-frame bursts in each hour of the experimental period (note that the K-L divergence ratio is plotted using a logarithm base 10 scale for ease of presentation).

Based on the results shown in Figure 15 and Figure 16, the three models were retrained using the trace data collected in the first 4 min of each hour in the experimental period. For each training process, three 4-min binary records were synthesized using the three error models, respectively. The authenticity of the resulting error models was then evaluated using a new performance metric *R*, defined as:

(3) $$R=\left|\frac{D_{m}}{D_{i}}\right|,$$

where *D_i_* is the K-L divergence of the independent model and *D_m_* is the K-L divergence of the two-state Markov model or second-order Markov model. In other words, a value of *R* less than 1 indicates that the Markov model (two-state or second-order) outperforms the independent model, and vice versa. Figure 17 shows the value of *R* for the correct-frame bursts in each hour of the experimental period. For the fourth and sixth hours, both Markov models outperform the independent model. Furthermore, the two models provide a comparable performance to the independent model in all of the other hours. It is additionally noted that the second-order Markov model significantly outperforms the two-state Markov model in the sixth hour. Figure 18 presents the equivalent performance results for the error-frame bursts. For ease of presentation, the *y*-axis is plotted with a base 10 logarithmic scale. Hence, a value of *R* less than 0 indicates that the Markov models outperform the independent model, and vice versa. The results show that the second-order Markov model generally outperforms the other two models, particularly in the third and seventh hour.

## 5. Overestimation Errors of Independent Model

According to the IEEE 802.15.4e standard, a data frame may be retransmitted three times at most. In other words, each data frame has a maximum of four transmission opportunities. Furthermore, after a transmitting node sends a data frame to a receiving node, the receiving node must return an ACK frame within a certain time interval. If the transmitting node receives the correct ACK frame within this interval, it deems the transmission of the data frame to have been a success. Otherwise, it performs retransmission up until the maximum number of allowable retransmissions. Assuming that the transmission quality of the wireless links can be modeled by the FDR, the transmission errors can be described by the original independent error model in OpenWSN. Let *P* denote the FDR from a transmitting node to a receiving node. The probability that the receiving node fails to correctly receive the data frame in the first transmission can therefore be written as:

(4) 1−P.

Accordingly, the probability that the receiving node fails to correctly receive the data frame for the first transmission and the three consecutive retransmissions can be written as:

(5) $${\left(1-P\right)}^{4}.$$

Excluding the above probability, the remaining probability is given as follows:

(6) $$1-{\left(1-P\right)}^{4},$$

where this probability represents the probability that the receiving node successfully receives the data frame within the maximum number of retransmissions. This probability can be used to represent the transmission reliability of a single wireless link. Note that due to the dynamic nature of the transmission quality in wireless networks, the probability, *P,* in the above formulae is unlikely to be a constant value.

OpenWSN adopts the average FDR computed over an extended time interval as the FDR for each moment. In other words, the independent error model assumes that all the frame delivery failure events are evenly dispersed over the considered time interval. However, in practical wireless networks, the frame transmission failures are more likely to burst in a short period, *T*. In other words, the transmission reliability of the wireless links is likely to be relatively low in the time interval *T*, but relatively high in all the other time intervals. As a result, the transmission reliability in OpenWSN simulations tends to be overestimated since the error frame burst lengths generated by the independent error model are very likely to be less than four.

For illustration purposes, assume that a transmitting node sends one data frame to a receiving node every minute for a total of 10 min, and in one of these minutes, the frame cannot be successfully delivered due to physical obstacles in the transmission path or the presence of interference, for example. Assume further that in the other 9 min, the data frames (9 in total) are successfully delivered without the need for retransmission. In other words, the overall average FDR is 9/(4 + 9) = 0.692. In this example, the transmitting node transmits a total of 10 data frames within 10 min, of which 1 data frame is not delivered due to poor wireless link conditions, but the other 9 data frames are successfully delivered. That is, the transmission reliability is equal to 0.9. By contrast, since the average FDR is 0.692, Equation (6) gives the transmission reliability as 1 − (1 − 0.692)^4^ = 0.991. In other words, the transmission reliability evaluated by the independent error model (0.991) overestimates the actual transmission reliability (0.9).

## 6. Simulation Results and Discussion

Simulations were performed to compare the performance of the second-order Markov error model proposed in the present study with the original independent error model in the OpenWSN simulator. The simulation environment consisted of two nodes, namely Node 1 serving as the gateway and Node 2 serving as the sensing device. The sensing device generated a 17-byte application-layer protocol data unit (APDU) every 3 s, where four of these bytes were serial numbers starting from the value of 1. In each experiment, Node 2 generated a total of 2000 distinct APDUs and sent them to Port Number 15001 of the gateway. Each time the gateway received a UDP packet with a destination port number of 15001, it added the packet to a text file for offline performance evaluation. The sensing node generated data for transmission only when it synchronized with at least one node in the OpenWSN network. It is noted that this approach is consistent with the general design of IWSN applications that repeatedly report the readings from a sensor. Since there is no datum generated during the period for which the sensing node is desynchronized from the network, the construction and maintenance of the IWSN has a trivial impact on the transmission reliability.

When the data frames carrying the above-mentioned APDU (or the corresponding ACK frames) were lost, the retransmission mechanism was automatically invoked. During retransmissions, the newly generated data frames cannot be sent immediately. In such a case, a buffer may increase the opportunities that the newly generated data frames be delivered later. However, in lossy environments, the buffer tends to be full, and hence the most-recently sensed data are simply dropped. Practical IWSNs generally prefer more recently-sensed data to older data since it is precisely the latest information on the plant state which is the most critical importance for most control applications [32]. However, the sensing nodes in IWSNs are usually resource-constrained and therefore cannot implement a buffering mechanism. As a result, the applications installed in IWSNs rarely allocate buffers for sensing data. In accordance with these observations, the testing application used in the present simulations also did not implement a buffering mechanism. That is, if the sensing node was still busy sending a previous data frame, the currently generated data frame was simply dropped. As a consequence, the error frame bursts not only affected the transmission reliability directly, but also impacted the transmission reliability indirectly through the frame drops produced during data retransmission.

Prior to performing the simulations, the trace acquired in the sixth hour of the experimental period was inspected to identify the 4 min segment having the poorest transmission quality. It was found that the records within the time interval from the 51st minute to the 54th minute had the lowest FDR (0.753). The transition probabilities *p*000, *p*010, *p*100 and *p*110 of the second-order Markov model trained using this segment were 0.860, 0.595, 0.746 and 0.379, respectively. In the subsequent simulation performed using the original independent error model in OpenWSN, Node 1 (the gateway) received a total of 1950 distinct APDUs. In other words, the transmission reliability was equal to 0.975 (i.e., 1950/2000 = 0.975). By contrast, in the simulation performed using the proposed second-order Markov error model, the gateway received a total of 1825 distinct APDUs. In other words, the transmission reliability was around 0.913. The level of overestimation of the transmission reliability was therefore reduced by (0.975 − 0.913) ÷ 0.913 = 0.0679, i.e., by around 6.8% in the considered scenario.

It is reasonable to assume that there exist industrial environments in which the transmission quality is poorer than that in the factory considered in the present study. For such environments, it is further reasonable to characterize the transmission reliability using the proposed second-order Markov model with transition probabilities *p*000, *p*010, *p*100 and *p*110, specified as fractions of the transition probabilities *p*000, *p*010, *p*100 and *p*110 trained by the records within the time interval extending from the 51st to 54th minute of the sixth hour in the measured experimental trace. Thus, four further simulations were performed with fractions of 0.9, 0.8, 0.7 and 0.6, respectively. The corresponding results are shown in Table 1, where the data in the first row correspond to the experiment described above, while the data in the second to fifth rows correspond to the simulations performed with fractions ranging from 0.9 to 0.6, respectively. For each simulation, the transmission reliability is denoted as *TR*. Furthermore, columns *Our TR* and *Original TR* show the transmission reliabilities derived by the simulator with the proposed second-order Markov model and the independent model, respectively. Finally, the column *Equation (6) TR* shows the transmission reliability computed using Equation (6).

It could be seen that the differences between the reliability results obtained from the independent error model and those derived from Equation (6) increase with a decreasing fraction. To investigate this phenomenon, we devised a particular function in the simulator for recording the frame drops produced during retransmission, and once again performed a simulation with FDR being 0.327, which is the same as the FDR in the fifth row of Table 1. The experimental results showed that 887 data frames were not received, and, among them, 606 data frames were dropped by the sender during data retransmission. In other words, only 1394 (i.e., 2000 − 606 = 1394) data frames are processed by the independent error model. Excluding the dropped frames, the transmission reliability is (2000 − 887) ÷ 1394 = 0.798, which is very close to the derived transmission reliability (i.e., 0.795) using Equation (6). Note that Equation (6) does not take into account the frame drops produced during data retransmission, which is the main reason for the inconsistency between the data in columns *Original TR* and *Equation (6) TR.*

In each simulation, a record was made of the total number of frames successfully received and the total number of transmissions. The corresponding FDR was then derived as the ratio of the former to the latter. Let the data in columns *Our TR* and *Original TR* be denoted as *x* and *y*, respectively. The accuracy improvement (AI) of the second-order Markov model over the original independent model can then be evaluated as:

(7) $$AI=\frac{y-x}{x}.$$

It is seen in Table 1 that the accuracy improvement obtained by the second-order Markov model increases with an increasing burst rate. Overall, the results show that the proposed model improves the accuracy of the transmission reliability estimates by 0.123 (see the third row in Table 1), for which the FDR is equal to approximately 0.5.

## 7. Conclusions

This paper has proposed a second-order Markov model for estimating the frame-level transmission errors in an industrial WSN based on the experimental traces obtained in a real-world factory environment over a ~24 h period. A statistical analysis has shown that the proposed model provides a better description of the transmission quality in industrial WSNs than conventional methods. In addition, the simulation results have shown that the proposed error model improves the accuracy of the estimated transmission reliability in the OpenWSN simulator by up to 12% compared to that achieved using the original independent error model. The performance advantage of the proposed model is particularly apparent when the transmission failure events are non-uniformly dispersed in a certain time interval.

## Figures and Tables

**Table 1:** Accuracy Improvements.

*Fraction*	*p*000	*p*010	*p*100	*p*110	*Our TR* ^1^	FDR	*Original TR*	*Equation (6) TR*	*AI* ^2^
1	0.860	0.595	0.746	0.379	0.913	0.753	0.975	0.996	0.068
0.9	0.774	0.536	0.671	0.341	0.820	0.618	0.892	0.979	0.088
0.8	0.688	0.476	0.596	0.303	0.716	0.515	0.804	0.945	0.123
0.7	0.602	0.417	0.522	0.265	0.606	0.415	0.667	0.883	0.100
0.6	0.516	0.357	0.447	0.227	0.494	0.327	0.546	0.795	0.105

^1^ TR denotes Transmission Reliability and ^2^ AI denotes Accuracy Improvement.
